# *Helicobacter pylori*: main cause of gastric adenocarcinoma in sub-saharan Africa, status report and control strategies

**DOI:** 10.1097/MS9.0000000000003376

**Published:** 2025-05-29

**Authors:** Christian Tague, Dujardin Makeda, Hermann Yokolo, Ekouo Joshua, Rodrigue Fikiri Bavurhe, Aymar Akilimali

**Affiliations:** a Department of Research, Medical Research Circle (MedReC), Bukavu, DR Congo; b Faculty of Medicine, Official University of Bukavu, Bukavu, DR Congo

*Dear Editor*,

*Helicobacter pylori* is a public health problem in sub-Saharan African countries and is a class I carcinogen. In sub-Saharan Africa, it represents a threat in the occurrence of gastric adenocarcinomas, and its infection varies considerably from one region to another. In West Africa, a study conducted in Ghana in 2017 and Nigeria in 2019 respectively revealed a prevalence of approximately 70% to 85% of the infected population. Also in East Africa, countries such as Kenya and Ethiopia have similar rates^[1]^. In Central Africa, the prevalence recorded in Chad in 2016 is approximately 70%; in 2018, the Democratic Republic of the Congo (DRC) recorded a rate of 85%, while in Sudan it is 75% in 2015^[2]^. This high rate of infection in these regions is due to high population density, promiscuity facilitating the transmission of the germ, also due to the low socio-economic situations of the populations, and the lack of hygiene^[3,4]^.

## The African enigma

However, despite this very high prevalence of *H. pylori* in sub-Saharan Africa, the incidence of gastric adenocarcinoma remains paradoxically low compared to other regions with similar infection rates. This phenomenon, commonly referred to as the African Enigma, has been widely described in the literature^[5,6]^. Several hypotheses have been advanced to explain this discrepancy, including the protective role of co-existing intestinal parasites, genetic differences in *H. pylori* strains, host genetic factors, and differences in dietary patterns. Further research is still needed to fully understand this phenomenon.

The prevalence of stomach cancer in 2019 in Nigeria and in Ethiopia in 2016 reveals that out of 100,000 infected inhabitants, 10–20 cases develop into stomach cancer. In Kenya in 2016, it reveals 6 to 10 cases per 100 000 infected inhabitants, and in Tanzania and Uganda respectively in 2015 and 2018, it reveals 9–12 cases per 100 000 infected inhabitants^[3,7]^. The risk of gastric cancer associated with *H. pylori* infection is higher in individuals over 50 years of age, because this infection, often acquired during childhood, can last a lifetime without treatment. In addition, men often have a greater vulnerability than women. Finally, a family history of gastric cancer and certain genetic predispositions, such as Lynch syndrome, which affects DNA repair, also contribute to increasing this susceptibility^[8]^.

A diet high in salt, smoked or salty foods, and low in fresh fruits and vegetables is associated with a higher risk of gastric cancer, as these habits can alter the gastric mucosa and promote carcinogenesis. In addition, excessive alcohol consumption and smoking are known risk factors, smoking being particularly associated with malignant tumors of the esophagus. Poor sanitation and contaminated drinking water are two important environmental factors that contribute to *H. pylori* infection, particularly in developing countries where sanitation conditions are poor^[9]^. Limited access to health care increases the risk of serious complications, such as gastric cancer, as affected populations are less likely to be diagnosed and treated for *H. pylori* infection. Moreover, poverty is often linked to poor nutrition and poor sanitation, which contributes to a higher incidence of *H. pylori* infections and, consequently, an increased risk of gastric cancer. Finally, low levels of education may influence awareness of the risks associated with *H. pylori* and healthy dietary practices, thereby affecting the prevalence of infections and their treatment^[10]^.

Prevention and control of *H. pylori* infection in sub-Saharan Africa requires a comprehensive and multidisciplinary approach. Primary prevention focuses on improving hygiene and sanitation practices among populations through the promotion of hand and food hygiene, and awareness campaigns to inform populations about the risks associated with consuming contaminated water and food^[8]^. Secondary prevention involves *H. pylori* screening programs, particularly for people with a family history of gastric diseases, which will allow the disease to be diagnosed at an early stage and thus reduce the incidence of *H. pylori*-related gastric adenocarcinomas. It is also wise to focus on the management of confirmed cases through triple or quadruple therapy with bismuth salt, which involves the rapid and effective use of antibiotics against *H. pylori* such as clarithromycin, amoxicillin, bismuth, and omeprazole. These actions aim to reduce serious complications such as gastric cancer. Tertiary prevention is based on the management of complications, particularly gastric adenocarcinoma; early management is synonymous with a good prognosis. Monitoring and treatment protocols for patients with a history of *H. pylori* infection should be established to manage long-term effects and prevent relapses^[7]^.

## Limitations

This article is based on a narrative synthesis of existing literature, and as such, does not present new clinical data. Additionally, variations in diagnostic methods, study populations, and health system capacities across sub-Saharan Africa may influence the comparability of prevalence and incidence data. The lack of large-scale, longitudinal studies in many African countries also limits the ability to draw strong conclusions about causal relationships between *H. pylori* and gastric cancer in this context.

In conclusion, *H. pylori* infection represents a crucial public health issue in sub-Saharan Africa, with serious implications for population health, notably the development of gastric adenocarcinoma. A multidisciplinary approach is essential to reduce the prevalence of this infection and its fatal consequences. In addition to public health strategies, further epidemiological and molecular research is needed to better understand the unique patterns of disease progression in this region, particularly in light of the African Enigma.

## Data Availability

Not applicable.
